# Degradation of Phenol via Meta Cleavage Pathway by *Pseudomonas fluorescens* PU1

**DOI:** 10.5402/2012/741820

**Published:** 2012-01-23

**Authors:** Md. Mahiudddin, A. N. M. Fakhruddin, Abdullah-Al-Mahin

**Affiliations:** ^1^Department of Environmental Sciences, Jahangirnagar University, Savar, Dhaka 1342, Bangladesh; ^2^Institute of Food and Radiation Biology, Atomic Energy Research Establishment, Savar, Dhaka 1342, Bangladesh

## Abstract

Degradation of phenolics by members of soil microflora is an important means by which these substances are removed from the environment thus reducing environmental pollution. Biodegradation by microorganisms offers unique opportunities to destroy or render phenolic compounds. A bacterium, PU1, identified as *Pseudomonas fluorescens* PU1, was investigated for its ability to grow on and degrade phenols as sole carbon sources in aerobic shaking batch culture. The organism degraded up to 1000 ppm of phenol using meta cleavage pathway. The pathways for phenol degradation were proposed by the identification of metabolites and assay of ring cleavage enzymes in cell extracts. Phenol was degraded via catechol with subsequent metaring cleavage. Cell growth increased as the phenol concentrations increased up to 1000 ppm phenol. The biodegradation efficiency, degradation extent, and metabolic pathway of phenol were determined to provide useful clues for further application of this isolate in the engineered bioremediation systems. The paper's results suggest that *Pseudomonas fluorescens* PU1 strain could be a good candidate for remediation of phenol contaminants from heavily polluted sites.

## 1. Introduction

Phenols are aromatic compounds that are characteristic pollutants in wastewater and effluents from chemicals, petrochemicals, pharmaceuticals, textiles, and steel industries [1]. The unwholesome and environmentally unacceptable pollution effects of the phenolic effluent have been reported worldwide [2]. Phenol contaminants are relatively soluble in water and accumulate in soil, resulting in extensive surface water, ground water, and soil contamination owing to its severe toxicity [3]. Currently removal of phenol effluents from contaminated sites has been a major environmental concern.

 Different techniques have been applied to remove phenolic compounds from polluted areas [4–8]. However, among all, biodegradation process offers the more opportunities to completely destroy the pollutants if possible or at least to transform them to innocuous substance [9], it posses relatively low cost, no chemicals used, and high public acceptance [10].

 Research on microbial degradation on phenols has intensified in recent years because it is the sustainable ways to clean-up contaminated environments [11]. Microbes will adapt quite rapidly and grow at extreme condition using hazardous compounds as carbon and energy sources, microbes can adapt rapidly to extreme conditions in waste streams. Important examples include phenol, chlorophenol, chlorobenzene, chloroalkanes, atrazine, and nitroaromatics [11].

A wide variety of microorganisms are known to be capable of metabolizing or mineralizing phenol under aerobic and/or anaerobic conditions. Metabolic processes are governed by the action of enzymes [12]. Many microbes belonging to the genus of *Pseudomonas* have been reported as good degraders of phenol. The pure culture of *Pseudomonas* strains are often utilized for metabolic pathway studies evaluating the degradation of many aromatic compounds such as phenol [13, 14]. In *Pseudomonas*, many induced enzymes are nonspecific, and the metabolic pathway contains a high degree of convergences. The convergence of catabolic pathway allows for the efficient utilization of a wide range of growth substrates, while the nonspecificity of the induced enzymes allows for the simultaneous utilization of several similar substrates without redundant genetic coding for enzyme induction [15].

**Figure 1:**
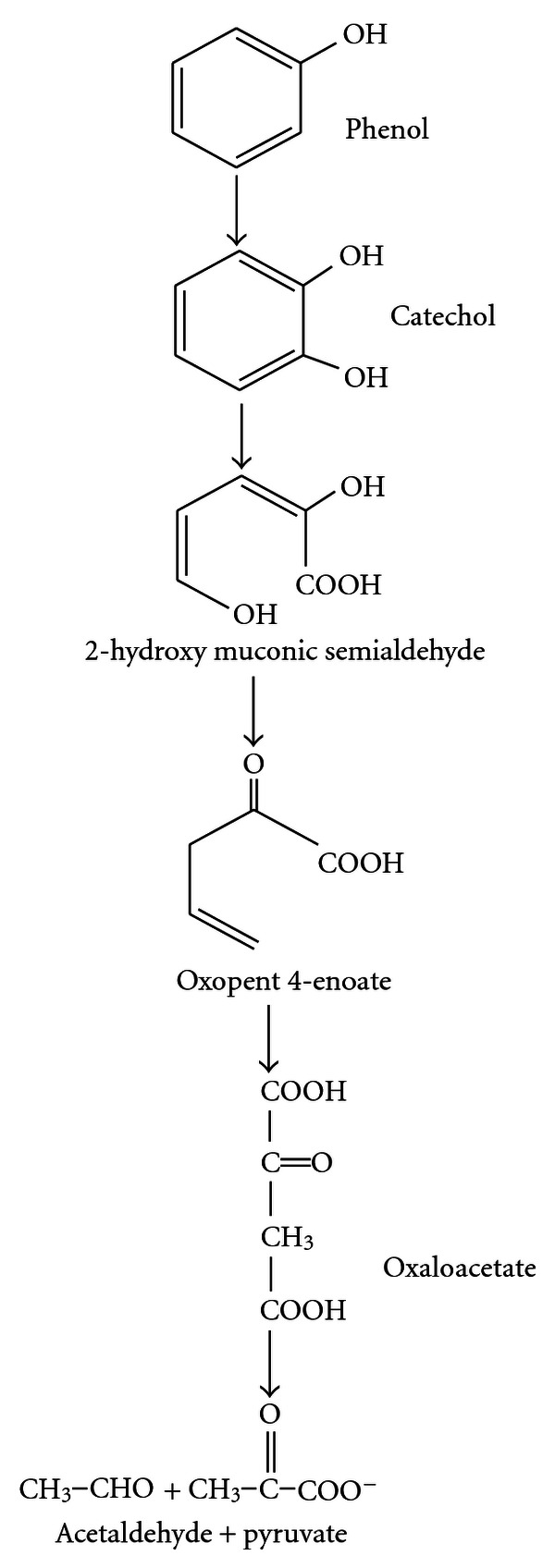
Metapathway of phenol degradation.

**Figure 2:**
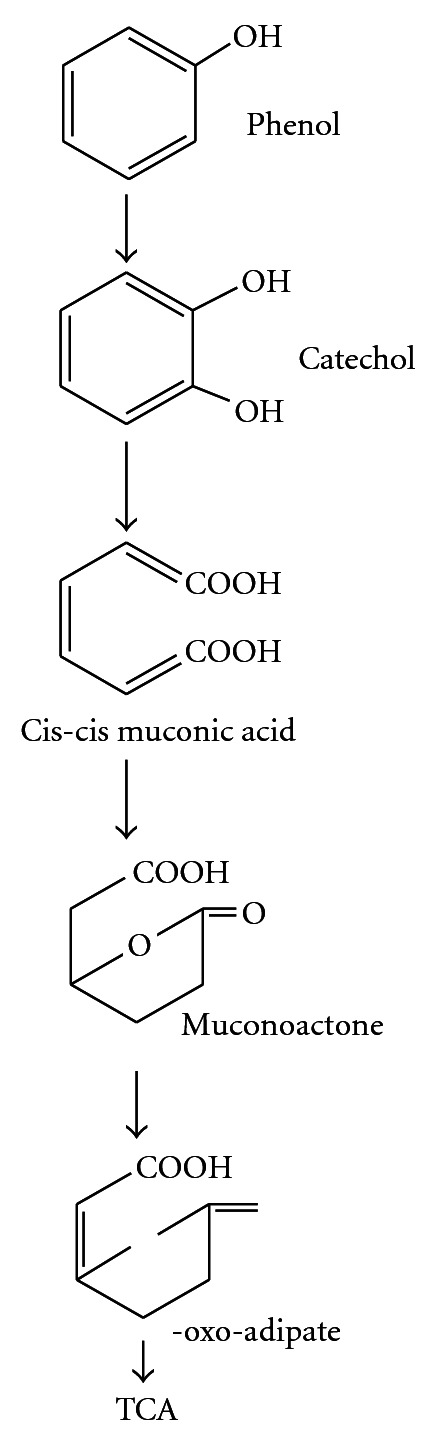
*Ortho*-pathway of phenol degradation.

A typical pathway for metabolizing an aromatic compound like phenol is to dihydroxylate the benzene ring to form a catechol derivative and then to open the ring through *ortho-* or metaoxidation. Catechol is oxidized via *ortho*-cleavage pathway by catechol 1,2-dioxygenase, or by metapathway to 2-hydroxymuconic semialdehyde by catechol 2,3-dioxygenase (Figures 1 and 2). The final products of both the pathways are molecules that can enter the tricarboxylic acid cycle [16, 17]. Catechols are cleaved either by *ortho*-fission (intradiol, i.e., carbon bond between two hydroxyl groups) or by a metafission (extra diol, i.e., between one of the hydroxyl groups and a nonhydroxylated carbon) as given in Figures 1 and 2. Thus the ring is opened and subsequently degraded [18].

In a biological treatment system, a potential strain is necessary for the effective degradation to proceed at a faster rate. Considering the potential of *Pseudomonas* strains, the present study was envisaged with the following objectives: isolation, screening, and identification of potential phenol degrading isolates and determination of phenol degradation pathway.

## 2. Materials and Methods

### 2.1. Culture Medium

The minimal medium used in the degradation studies, adapted from Goulding et al. [19], contained (g/L) K_2_HPO_4_, 4.36; NaH_2_PO_4, _3.45; NH_4_Cl, 1.0; MgSO_4_·6H_2_O, 0.912; trace salts solution 1 mL/L. The trace salts solution was prepared separately in distilled water and was stored in a dark bottle for 6–8 weeks. The trace salts solution contained (g/100 mL) CaCl_2_·2H_2_O, 4.77; FeSO_4_·7H_2_O, 0.37; CoCl_2_·6H_2_O, 0.37; MnCl_2_·4H_2_O, 0.10; Na_2_MoO_4_·2H_2_O, 0.02. The pH of the medium was adjusted to 7.0 with 2 M NaOH. Phenol was added to the minimal medium after sterilization. Minimal media together with phenol was used for biodegradation studies.

### 2.2. Isolation and Screening of Phenol-Degrading Bacteria

The soil samples were collected aseptically from different sites under three inches of depth from the surface soil. The samples were taken in presterilized McCartney bottle and capped air tightly. One gram of soil was suspended in 9.0 mL sterile distilled water, agitated for mixed well. After dilution, 0.1 mL suspension was spread over *Pseudomonas* minimal agar plates (pH 7 ± 0.1) containing 200 ppm of phenol as sole carbon and energy source. Spread plate techniques as described by APHA [20] were followed in our study. All plates were incubated for 24–48 h at 30°C. A number of bacterial colonies showing on plates were selected, streaked twice on *Pseudomonas* minimal agar plates by replica plate method [21] for purification. When a streaking produced only one type of colony in a plate, it was considered to be pure culture [22]. For obtaining high potential isolates a preliminary screening was done employing *Pseudomonas* minimal agar plates with 500 ppm concentration of phenol. Among the five high tolerant bacteria, further secondary screening was conducted applying 800 ppm of phenol in liquid media. Five microbial strains (designated as PU1, PK2, PK3, PK4, and PF6) obtained as described above were maintained as pure culture over minimal agar slants at 4°C for further studies. Thus the most tolerant isolate was finally characterized on the basis of morphological, cultural, and biochemical properties [23–25].

### 2.3. Identification of the Isolate

The selected bacterial isolate PU1 was identified by morphological and biochemical characterization as per Bergey's Manual of Systematic Bacteriology [26, 27]. Bergey's Manual of Determinative of Bacteriology [28] and “ABIS6” online software (accessed on 20 January 2011) [29] were used as a reference to identify the isolate.

### 2.4. Cultural Conditions

Isolate PU1 was used to inoculate in nutrient broth (1.3%, w/v) and incubated at 37°C for 24 h with agitation at 120 rpm. The harvested cells were centrifuged at 5000 rpm for 10 minutes and washed twice with 0.01 M sodium phosphate buffer and final pellet resuspended in the same buffer. Five mL of bacterial suspension (*≈*10^6^ to 10^8^) [19] was used to inoculate 95 mL sterile minimal medium containing appropriate phenol concentration in 250 mL conical flasks. Media was sterilized by autoclaving prior to the addition of phenol. After inoculation, flasks were incubated in an orbital shaker at 120 rpm at 37°C. Samples were aseptically removed at regular intervals and analyzed for cell growth and phenol removal. Samples were aseptically taken for biomass and phenol assay. Cells were removed by centrifugation at 5000 rpm for 10 minutes and the supernatants were analyzed for phenol removal.

### 2.5. Measurement of Growth of the Organisms

Growth was monitored by using optical density measurement at 660 nm (OD_660_) [30] using UV-spectrophotometer (Shimadzu 1601).

### 2.6. Chemical Analysis

Phenol concentrations were determined by using the 4-aminoantipyrene colorimetric method based on the procedure detailed in Standard Methods for the Examination of Water and Wastewater [31].

### 2.7. Enzyme Assay

#### 2.7.1. Preparation of Cell-Free Extract

Cells were grown on phenol (600 ppm) which was harvested by centrifugation (4000 rpm, 10 min) and the resulting pellet was washed twice with 0.33 M Tris-HCl buffer (pH 7.6). The cells were broken by sonication for 4 minutes (30 sec on, 30 sec off) and centrifuged at 12000 rpm, 4°C for 20 min. The cell-free extract was kept on ice and assayed as soon as possible for catechol dioxygenase activity using the method of Feist and Hegeman [32].

#### 2.7.2. Catechol 1,2-Dioxygenase Activity

The *ortho*-cleavage product of catechol is catechol 1,2-dioxygenase it was measured by following the formation of cis,cis-muconic acid. The following reagents were added to a quartz cuvette: 2 mL of 50 mM Tris-HCl buffer (pH 8.0); 0.7 mL of distilled water; 0.1 mL of 100 mM 2-mercaptoethanol, and 0.1 mL of cell-free extract. The contents of the cuvette were mixed by inversion and 0.1 mL of catechol (1 mM) was then added and the contents mixed again. The absorbance read at 260 nm over a period of 5 min and cis,cis-muconic acid formation was indicated by the increase of absorbance.

#### 2.7.3. Catechol 2,3-Dioxygenase Activity

Catechol 2,3-dioxygenase activity was measured by following the formation of 2-hydroxymuconic semialdehyde, the meta cleavage product of catechol. The following reagents were added to plastic cuvette: 2 mL of 50 mM Tris-HCl buffer (pH 7.5), 0.6 mL of distilled water, and 0.2 mL of cell-free extract. The contents were mixed by inversion and 0.2 mL of catechol (100 mM) was added and mixed with the contents. 2-Hydroxymuconic semialdehyde production was followed by an increase in absorbance at 375 nm over a period of 5 min.

#### 2.7.4. Calculation of Enzyme Activity

The enzyme activity was calculated by using the following equation:

(1) $$\begin{array}{cc} & \text{Activity}\;\left(\mu \;\text{moles}\;\text{product}\;\text{formed/min}\right)\\ & \;\;=\;\frac{\text{E}\times \text{C}\times \text{L}}{\text{V}}\times \frac{\Delta \text{OD}}{\min}. \end{array}\;$$

Molar extinction coefficient for catechol 1,2-dioxygenase, E_260_ = 16,800 L/mol/cm and for catechol 2,3-dioxygenase, E_375_ = 14,700 mol/L/cm.

Specific activities were expressed as units per milligram of protein calculated by the following equation:

(2) $$\begin{array}{cc} \text{Specific}\;\text{activity}\;\left(\mu \;\text{moles/min/mg}\right)\; & =\;\frac{\text{Activity}}{\text{Total}\;\text{protein}} \end{array}.$$

The protein concentrations in cell-free extracts were determined by the method of Lowry et al., with bovine serum albumin as the standard [33].

### 2.8. Statistical Analysis

The experimental data was analyzed using Sigma plot 7 (2001) and Microsoft Office Excel 2007.

## 3. Result and Discussion

### 3.1. Isolation and Screening of Phenol-Degrading Bacterial Cultures

Phenol-degrading bacteria were isolated from the soils adjacent to the plant roots. Sample soils were cultivated on *Pseudomonas* minimal media containing phenol as sole carbon and energy source at 37°C. Five phenotypically different colonies were picked from the plates and translated to fresh pseudomonas minimal agar plates with phenol for purification [34].

### 3.2. Screening

**Figure 3:**
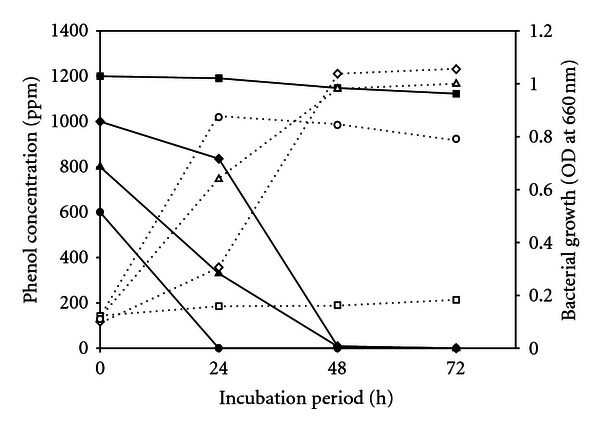
Removal of phenol at different concentration and corresponding cell growth by the isolate *Pseudomonas fluorescens* PU1 (blocked lines indicate removal and dotted line indicate growth).

As many as five bacterial isolates were purified and inoculated to *Pseudomonas* minimal medium containing phenol (800 ppm) as the sole carbon and energy source. The growth of the bacteria in terms of absorbance, and the extent of phenol degradation were monitored up to 3 days. It could be seen that the biomass growth and phenol removal by four isolate PU1 was higher than other four isolates. Hence, the results of phenol degradations were reported up to 3 days (Figure 3).

### 3.3. Morphological and Biochemical Characteristics of Isolate PU1

The isolate PU1 was short rod and round in shape, stained gram negative, and was positive for catalase and oxydase activity. It gave negative result for methyl red and indole test. Isolate PU1 also gave positive result for the Voges-Proskauer test and was able to hydrolyze gelatin. The strain utilized sugar glucose, fructose, sucrose, xylose, and sorbitol, but did not utilize rhamnose, arabinose, and lactose. Based on the morphological, biochemical, and carbohydrate utilization tests, it was identified as *Pseudomonas fluorescens* [28, 29].

### 3.4. Removal of Phenol and Growth Rate of Phenol-Degrading Bacteria

The highest tolerance level of strains PU1 was determined. This strain was allowed to grow for 72 hours, in the *Pseudomonas* minimal medium containing phenol at different concentration as the sole source of carbon and energy. This isolate completely degraded phenol up to 600 ppm in 24 hours and corresponding bacterial cell growth (i.e., OD at 660 nm) was 0.873 at 24 hours from the initial cell density 0.110. After 24 hours the cell density was recorded to be decreased with time (Figure 3) due to absence of carbon source.

**Figure 4:**
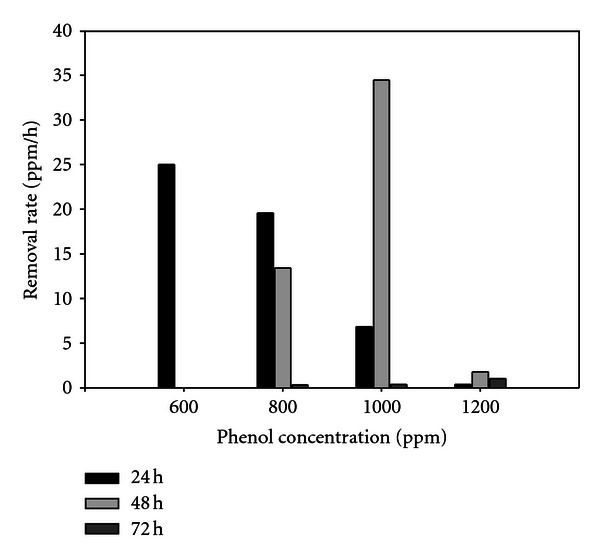
Removal rate of phenol at various concentrations by *P. fluorescens *PU1 when supplied as the sole source of carbon and energy.

*Pseudomonas fluorescens *PU1 also completely degraded 800 ppm and 1000 ppm of phenol in 72 hours whereas it degrades about 99 percent of phenol in 48 hours as well. The highest cell growth was found at 72 hours for 800 ppm and 1000 ppm of phenol as 0.999 and 1.055, respectively. It was observed that in case of 1200 ppm of phenol, only 6.45 percent phenol was removed by our isolate in 72 hours of incubation. There was no significant enhancement in cell density in medium containing 1200 ppm of phenol. In the present study, pH of the medium was considered to be 6.9 ± 0.1 at 37°C. The removal rate of phenol at different concentration by the isolate was observed, and it is significant in the sense that phenol removal rate was directly related to increased cell growth (Figure 4).

A number of phenol-degrading aerobic bacteria have been described previously by other researchers [35–38]. The concentration of phenol and presence of halogenated substrates seem to play a crucial role on degradation shown in our study and also reported by others. High concentrations of phenol are usually inhibitory to growth of organisms [35].

Compared to four isolate, *Pseudomonas fluorescens* PU1 seems to be superior in terms of resistance or tolerance to phenol, since it could tolerate phenol up to the concentrations of 1000 ppm. Hence, the present study has demonstrated that the *Pseudomonas fluorescens *PU1 could play an important role in the remediation of phenolics in heavily polluted sites.

### 3.5. Analysis of Enzyme Activity of the Organisms

Enzyme activity was determined for the organism grown on phenol because growth substrate can influence the enzyme produced. Catechol is the common intermediate in aromatic degradation and can be metabolized via either the *ortho* or meta cleavage pathways [39].

The efficiency of a certain catabolic pathway often depends on the properties of the involved key enzyme(s). Therefore, the specific activities of phenol hydroxylase, catechol 1,2-dioxygenase, and catechol 2,3-dioxygenase in cell-free extracts obtained by sonication from investigated isolate was examined.

Analysis of intercellular enzyme activity indicated that *Pseudomonas fluorescens *PU1 showed greater metaactivity than *ortho*-activity which demonstrate degradation occurred using the meta cleavage pathway. Specific enzyme activity carried out following growth on phenol confirmed this (Table 1). Catechol 2,3-dioxygenase activity towards a number of catechol was induced in cell grown on phenol. No identifiable activity of catechol 1,2-dioxygenase was found.

After reviewing various literatures on the already established, it was found that phenol degraded in both *ortho* and meta cleavage pathway although *ortho-*pathway is most common [39–42].

Successful metabolism of phenol appears to require the meta cleavage pathway. Assays of the key enzymes involved in the ring cleavage of catechol 1,2-dioxygenase and catechol 2,3-dioxygenase indicated that degradation of the phenol *P. fluorescens *PU1 was via the meta cleavage pathway. Cells grown on phenol displayed greater metaactivities mainly towards catechol, while *ortho*-activity was very low. This suggests that *P. fluorescens *PU1 possesses meta cleavage enzyme, a catechol 2,3-dioxygenase capable of metabolising catechol.

## 4. Conclusions

The aerobic phenol-degrading isolate *Pseudomonas fluorescens *PU1 appears to have greater potential for enhanced phenol removal through utilization of phenol as sole source of carbon and energy. Resistance against a high concentration of phenol facilitates its use for biological treatment system of wastewater. Complete degradation of such a high concentration of phenol (1000 ppm) by metapathway is not well demonstrated previously. Here we report these isolate capable of growth at relatively high phenol concentrations together with the analysis of functional properties relevant to the application of this organism to the biodegradation of aromatic wastes (phenolics).

## Figures and Tables

**Table 1:** Enzyme activities in cell extracts during the degradation of phenol by *Pseudomonas fluorescens* PU1.

Enzyme assayed	Activity, U	Specific activity (U/mg)
Catechol 1,2-dioxygenase	3 × 10^−3^	0.0090
Catechol 2,3-dioxygenase	0.1417	0.429

Specific enzyme activities are expressed in micromoles per minute per milligram of protein.
